# A Review of Therapeutic Approaches for Autism Spectrum Disorder

**DOI:** 10.3390/brainsci15121280

**Published:** 2025-11-28

**Authors:** Yang Hai, Saihan Bai, Huiting Qiao, Deyu Li, Daifa Wang, Meiyun Xia

**Affiliations:** 1 Medical Engineering & Engineering Medicine Innovation Center, Hangzhou International Innovation Institute, Beihang University, Hangzhou 311115, China; 2 School of Biological Science and Medical Engineering, Beihang University, Beijing 100191, China

**Keywords:** autism spectrum disorder, pharmacological treatment, behavioral intervention, traditional Chinese medicine, neuro-modulation techniques, complementary and alternative medicine, precision medicine, early intervention, multi-modal therapy

## Abstract

Autism spectrum disorder (ASD) is a highly heterogeneous neurodevelopmental disorder. Significant progress has been made in the intervention and treatment of ASD. This review systematically summarizes five major categories of mainstream ASD treatment approaches. This article outlines the theoretical basis and therapeutic effects of each intervention method, discusses their advantages and limitations, and analyzes and forecasts future development directions. Due to the lack of specific treatment methods, ASD treatment primarily relies on behavioral interventions, supplemented by symptomatic pharmacological treatments. Behavioral interventions can significantly improve children’s self-care abilities and quality of life while also promoting social skills and communication, and reducing disability and comorbidity rates. ASD intervention methods should primarily focus on those proven effective through evidence-based practice, adhering to individualized, multidimensional, and multidisciplinary approaches, thereby promoting the development and establishment of efficient and personalized intervention strategies.

## 1. Introduction

Autism spectrum disorder (ASD) is a highly heterogeneous neurodevelopmental disorder characterized by early onset, a long course, and high disability rates [1]. Approximately one in every 100 children is diagnosed with ASD, which imposes a significant economic burden on families and society [2]. ASD rarely presents in a pure form and is often comorbid with psychiatric disorders [3], such as attention deficit hyperactivity disorder (28%), anxiety disorders (20%), repetitive behaviors and obsessive compulsive disorder (9%), sleep disorders (13%), and mood disorders (with a comorbidity rate of 11% for major depressive disorder and 5% for bipolar disorder). Neurological comorbidities such as cerebral palsy and epilepsy are also observed [4]. A cross-sectional study revealed that 65% of patients have comorbidities, including epilepsy (2%) and cerebral palsy (2%) [5]. Therefore, there is an urgent need for scientifically validated early intervention models.

**Figure 1:**
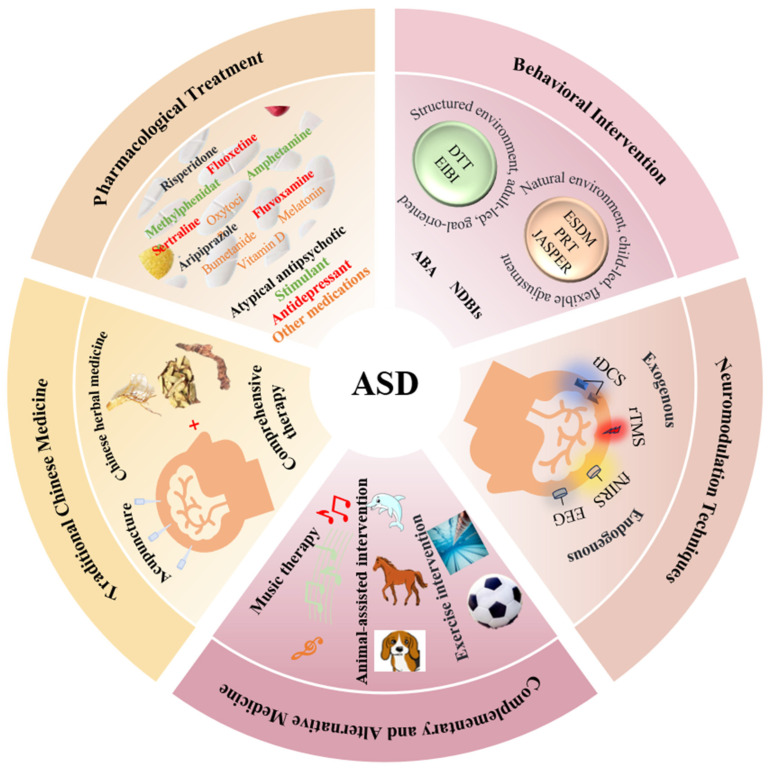
ASD intervention and treatment methods.

Currently, common treatment methods for ASD include pharmacological treatment, behavioral intervention, traditional Chinese medicine (TCM), neuromodulation techniques, and complementary and alternative medicine (CAM), as shown in Figure 1. Each method has its unique advantages. For example, pharmacological treatment is mainly used to manage comorbid symptoms related to ASD [6]. Behavioral intervention aims to improve social skills and adaptive behaviors [7]. TCM aims to address the root causes and symptoms of the disorder [8]. Neuromodulation techniques alleviate ASD-related symptoms by regulating brain activity [9,10]. CAM provides more options for personalized care.

This article systematically summarizes and discusses the theoretical basis, therapeutic effects, advantages, limitations, and future development directions of various methods. This review provides a comprehensive overview of current ASD interventions. It aims to serve as a comprehensive reference for clinical practice and future research, thereby promoting the development and establishment of efficient, personalized intervention strategies.

## 2. Pharmacological Treatment

Pharmacological treatment alleviates and manages comorbid symptoms of ASD by modulating neurotransmitters, emotional states, and behaviors [6,11,12]. Based on their mechanisms of action, pharmacological treatment can be broadly categorized into atypical antipsychotics [6,13,14,15,16,17], stimulants [18,19,20,21], antidepressants [22,23,24,25], and other potential therapeutic drugs [26,27,28,29,30,31].

### 2.1. Atypical Antipsychotic

Atypical antipsychotics aim to regulate the balance of neurotransmitters such as dopamine and serotonin, thereby reducing irritability and impulsive behaviors in patients [13,14]. Risperidone and aripiprazole are two atypical antipsychotics approved by the United States Food and Drug Administration [6,15,16,17]. Specifically, risperidone is suitable for ASD patients with high aggression and irritability, and can control severe behavioral problems in the short-term. Due to its good tolerability and lower incidence of metabolic side effects, aripiprazole is more suitable for patients in need of long-term management [32].

Studies have confirmed the efficacy of risperidone and aripiprazole in alleviating ASD symptoms. McCracken et al. conducted an 8-week randomized, double-blind, placebo-controlled trial to evaluate the positive effects of risperidone [33]. After treatment, the irritability scores in the risperidone group significantly improved, with a higher positive response rate compared to the placebo group. In some patients, the effects lasted nearly six months. Similarly, Marcus et al. assessed the efficacy of different doses of aripiprazole in alleviating irritability symptoms in adolescents with ASD [34]. The study found that all tested safe doses were significantly better than the placebo.

Newer atypical antipsychotics (brexpiprazole [35,36], cariprazine [37,38], lurasidone [39], etc.) have gradually been developed and applied [40,41,42], and their efficacy needs further investigation. For example, Yeung et al. evaluated the safety and tolerability of cariprazine in children with ASD [37]. The pharmacokinetic characteristics of cariprazine and its metabolites in children with ASD were characterized at doses of 3 mg QD (ages 13–17) and 1.5 mg QD (ages 5–12). Overall, cariprazine treatment was well tolerated, providing a basis for selecting appropriate pediatric doses in subsequent studies. However, related research has shown that brexpiprazole did not demonstrate significant efficacy in treating irritability associated with ASD [43].

In summary, atypical antipsychotics are effective and fast-acting in behavior management, providing a crucial time window for implementing other interventions [44]. However, their efficacy in addressing the core symptoms of ASD is limited, and they come with certain side effects, such as weight gain and drowsiness. Long-term use may lead to metabolic syndrome. Some medications may also increase the risk of arrhythmia, potentially leading to sudden cardiac arrest [45].

### 2.2. Stimulant

Stimulant medications work by increasing levels of dopamine and norepinephrine to improve attention and impulse control [18,19]. Studies have shown that approximately 30% to 50% of ASD patients may also exhibit symptoms of attention deficit/hyperactivity disorder (ADHD), such as impulsivity, inattention, and hyperactivity [46,47]. Therefore, stimulant medications are sometimes used to alleviate related symptoms in ASD patients [20,48].

In clinical practice, commonly used stimulant medications include methylphenidate and amphetamine-based drugs. Sturman et al. conducted a systematic review on the efficacy of methylphenidate in ASD patients aged 6 to 18 years [21]. They found that methylphenidate has a certain efficacy in alleviating symptoms of inattention, impulsivity, and hyperactivity, but it does not have a significant impact on the core symptoms of ASD. Additionally, stimulant medications may cause serious side effects, including appetite loss, sleep disturbances, and irritability [20]. Nevertheless, stimulants remain an important option for treating ADHD symptoms in individuals with ASD [6].

### 2.3. Antidepressant

Antidepressants primarily alleviate symptoms of depression and anxiety by increasing serotonin levels [22,23]. Selective serotonin reuptake inhibitors (SSRIs) are the most commonly used class of antidepressants in clinical practice [49]. Commonly used SSRIs include sertraline, fluoxetine, and fluvoxamine [24,25].

Steingard et al. demonstrated that short-term treatment with sertraline may reduce behavioral responses during situational transitions in children with ASD [50]. Hollander et al. [23] and Reddihough et al. [51] respectively confirmed that fluoxetine can reduce repetitive and compulsive behaviors in ASD patients. McDougle et al. found that fluvoxamine has certain efficacy in reducing perseverative thoughts and behaviors and alleviating aggression behaviors [52]. Similarly, the adverse effects caused by SSRIs are noteworthy, such as sleep disturbances, gastrointestinal discomfort [53], and in some cases, potential worsening of behavior.

Newer antidepressant (venlafaxine, desvenlafaxine [24], vortioxetine [54], etc.) have gradually been developed. Carminati et al. [55] objectively assessed the efficacy of low-dose venlafaxine treatment on behavioral improvement in adults with ASD through a randomized double-blind study. Principal component analysis revealed statistically significant differences between the venlafaxine group and the placebo group. This result statistically confirms that venlafaxine provides a new pharmacological avenue for addressing behavioral disorders in patients with intellectual disabilities and ASD.

### 2.4. Neuroendocrinological Therapies

Neuroendocrine products have also been found to have the potential to improve symptoms of ASD. For example, oxytocin can enhance social interactions and reduce repetitive behaviors in individuals with ASD [26,27]. Bumetanide [28], by indirectly enhancing the inhibitory effects of γ-aminobutyric acid, may reduce repetitive behaviors and improve emotion recognition [56,57,58]. Cannabidiol has shown improvements in behavior and social communication for ASD [29,30]. Hormonal medications can improve repetitive behaviors and hyperactivity [31]. Additionally, glutamate-related medications, by regulating the balance of neural excitation and inhibition, have some efficacy in improving social behavior [59,60,61,62]. Nutritional supplements, such as vitamin D [63,64] and melatonin [65,66], are believed to have auxiliary effects in improving sleep, behavior regulation, and cognitive function. Although these medications have shown some therapeutic potential, they are still in the experimental validation stage and have not yet been officially approved for treatment. Therefore, their efficacy still requires further validation through high-quality randomized controlled trials [48].

### 2.5. Advantages, Limitations, and Development Directions

Pharmacological treatment alleviates and manages comorbid symptoms of ASD by modulating neurotransmitters, emotional states, and behaviors [6,11,12]. This approach works quickly and is suitable for individuals with severe behavioral issues. However, it has limited effectiveness in improving the core symptoms of ASD and presents potential side effects [20,45,53] (See Table 1). Future research could focus on developing new targeted therapies, such as neurotransmitter-targeting drugs, and longitudinal follow-up studies to assess their long-term impact on core symptoms and disease progression. Additionally, there should be a systematic monitoring of adverse drug reactions, development of risk prediction models, and exploration of personalized dosage delivery models to minimize individual side effects.

## 3. Behavioral Intervention

Behavioral interventions aim to improve social skills and reduce problematic behaviors in individuals with ASD by altering behavior patterns. It primarily includes two approaches: applied behavior analysis (ABA) and naturalistic developmental behavioral interventions (NDBIs).

### 3.1. Applied Behavior Analysis

ABA emphasizes intervention in a structured environment, adult-led, with clear goals, focusing on behavior improvement, and is suitable for all age groups [143]. Its representative techniques include discrete trial training (DTT) and early intensive behavioral intervention (EIBI). DTT is a highly structured teaching strategy that breaks down target skills into small units and teaches them through repeated trials. EIBI, on the other hand, is a more comprehensive intervention approach that aims to enhance the overall functioning of children with ASD through early intensive intervention [67]. Existing research indicates that ABA has significant efficacy in improving social skills, language development, learning abilities, and adaptive behaviors in individuals with ASD [68,69,70]. However, the high-intensity training involved may impose a substantial burden on families, making it challenging to widely promote and implement on a large scale [71,72,73,74].

### 3.2. Naturalistic Developmental Behavioral Interventions

NDBIs focus on intervention in a natural environment, child-led, with an emphasis on improvements in social-emotional skills, and are often used during early childhood [7,144]. Representative methods include the early start Denver model (ESDM) [145,146], pivotal response treatment (PRT) [147,148], and joint attention symbolic play engagement and regulation (JASPER) [149,150]. Among these, ESDM focuses on individualized intervention and parental involvement [75]. PRT targets key developmental areas, using natural reinforcement strategies to promote the development of language and social skills [76]. JASPER focuses on enhancing joint attention and play skills in children with ASD [77].

Related research confirms that NDBIs have positive effects on language, cognitive, social communication, and adaptive behaviors in children with ASD [78,79]. For example, Dawson et al. conducted a two-year randomized controlled trial to evaluate the positive effects of ESDM on children with ASD aged 18 to 30 months [80]. The study found significant improvements in the combined standard scores on the Mullen scales of early learning and the vineland adaptive behavior scales in the ESDM group, indicating that ESDM can significantly improve cognitive function, adaptive behavior, and diagnostic severity in young children with ASD. Hardan et al. compared the effectiveness of PRT and psychoeducation in improving language impairments in children with ASD [81]. The study found that the PRT group showed significant improvements in observational measures compared to the psychoeducation group. This result indicates that PRT interventions can promote skill acquisition in functional and adaptive communication for both parents and children. Kasari et al. compared the effects of JASPER and psychoeducation on children with ASD aged 22 to 36 months. The results showed that JASPER, which targets core deficits, can sustainably improve social interaction skills in children with ASD [82].

It is undeniable that the above studies are based on small samples and have short follow-up periods, and the long-term efficacy of NDBIs requires further in-depth investigation [79,151]. Additionally, the involvement of families and communities is crucial, which is influenced by cultural and socioeconomic factors, presenting significant challenges for implementation in low- and middle-income countries [152,153].

### 3.3. Advantages, Limitations, and Development Directions

Behavioral interventions provide structured training protocols that can be adjusted according to individual needs, serving as the cornerstone of early intervention [7,143,144]. Behavioral interventions require long-term participation from the affected children, making them difficult to implement in resource-limited environments. Their effectiveness is highly dependent on the professional level of the therapists and the intensity of the intervention, with significant variability in individual outcomes. For example, the high repetitiveness and structured training in ABA may lead to resistance behaviors in children [71,72,73,74]. Future efforts should aim to develop low-cost, scalable intervention models, such as parent-led or community-supported interventions, to improve accessibility in resource-limited settings [152,153]. The integration of AI-assisted interventions and remote guidance systems may help standardize implementation while identifying key factors contributing to individual treatment response variability.

## 4. Traditional Chinese Medicine

TCM believes that the main cause of ASD is brain dysfunction, and it is closely related to the functions of the heart, liver, spleen, and kidneys [154,155]. TCM emphasizes the concept of entirety and individual treatment. In clinical research on ASD, TCM treatments mainly include acupuncture [83,84,85,156], Chinese herbal medicine [8,94,96,157,158,159], and comprehensive therapy [97,98,99]. Acupuncture involves inserting fine needles at specific angles into designated acupoints and manipulating them in certain ways to stimulate meridians and achieve therapeutic effects [8,160]. Chinese herbal medicine treatment is usually based on syndrome differentiation and includes patterns such as liver dysfunction and heart-spleen deficiency [8]. Comprehensive therapy integrates multiple TCM treatment methods to synergistically enhance the therapeutic effects on ASD [161].

### 4.1. Acupuncture

Acupuncture therapy for ASD includes scalp acupuncture, body acupuncture, and ear acupuncture, and other acupoint selection therapies based on individualized diagnosis. Among these, scalp acupuncture is the most widely used [83,84], with Jin’s three-needle technique being representative [85].

Yuan et al. compared the efficacy of Jin’s three-needle acupuncture and behavioral intervention in the treatment of children with severe autism [86]. The Jin’s three-needle group (*n* = 35) received acupuncture treatment with needles retained for 30–60 min once daily, six times a week, for 40 weeks. The behavioral intervention group (*n* = 34) received comprehensive intervention therapy. Results showed significant improvements in the total scores on the childhood autism rating scale (CARS) in both groups after treatment, but Jin’s three-needle group exhibited greater improvement, shorter onset time, and a significantly higher effective rate of 97.1%. This study indicates that Jin’s three-needle acupuncture can effectively improve symptoms in children with severe autism, with rapid onset and high efficiency. Wong et al. evaluated the efficacy of electroacupuncture [87]. Compared to the sham electroacupuncture group, the treatment group showed significant improvements on multiple autism assessment scales. In addition, other registered studies related to acupuncture techniques have been gradually initiated [4]. Some studies are currently ongoing (registration numbers: ChiCTR2100047559, ChiCTR2000029357, ChiCTR1900023247, ChiCTRINR-17012642, ChiCTR-IPR-17010558, ChiCTR-IOR-16010252), while some studies have been completed (registration numbers: ChiCTR2200056901, NCT00935701, NCT00352352, NCT00352248, NCT00355329, NCT00346736), and the results have not yet been reported.

Relevant animal experiment results indicate that certain treatments can improve the behavioral performance of ASD model rats. For instance, Jia et al. [88] found that Yu-Mu-Tiao-Shen acupuncture can regulate the expression of related proteins in autism model rats, modulate synaptic plasticity in the hippocampus and prefrontal neurons, and effectively enhance motor, social cognition, and spatial memory abilities in ASD rats. Functional imaging studies confirmed that acupuncture specifically activates the left hippocampus and bilateral pons, enhancing the functional connectivity between the parietal and frontal lobes, thereby improving language cognition abilities [89]. Acupuncture improves autism-like behaviors by repairing synaptic function of prefrontal neurons and regulating immune inflammation [90].

Most of the current research employs three or more scales for a comprehensive assessment of the results [162]. Although these scales effectively integrate information from patients, parents, and doctors, they are still insufficient to support the efficacy of acupuncture in the treatment of ASD. Relevant studies provide objective evidence for the effectiveness of acupuncture in the treatment of ASD. For example, Zhang et al. [91] measured plasma levels of AVP and OXT in patients post-treatment, which helps explore the potential mechanisms of acupuncture in ASD, particularly concerning the relevant acupoints from a modern medical perspective. Additionally, Zhao et al. [92] utilized SPECT to evaluate the efficacy of acupuncture in ASD, while Chan et al. [93] used quantitative electroencephalography to measure neurophysiological changes in patients to assess the effectiveness of acupuncture in ASD.

Acupuncture has shown considerable potential in improving autism symptoms, but its limitations cannot be ignored. Acupuncture is often accompanied by pain and fear, leading to low cooperation from children and an increased risk of adverse events, such as needle breakage. Electroacupuncture has a strong stimulation intensity and is not suitable for patients with arrhythmias or epilepsy. Additionally, acupuncture requires practitioners to possess high professional skills, and there is no standardized acupuncture protocol yet [156].

### 4.2. Chinese Herbal Medicine

Chinese herbal medicine exerts therapeutic effects on different types and stages of autism through synergistic regulation involving multiple components, multiple targets, and multiple pathways [158]. Commonly used medicines include Poria, Acorus calamus, Ginseng, Licorice, and Angelica, among others [94,95].

Cao et al. [96] conducted a nearly four-month controlled trial to evaluate the efficacy of Yangxin Kangpi decoction combined with intervention training in treating children with autism characterized by heart-spleen deficiency. The results showed significant improvement in the CARS scores of the patients. Chinese herbal medicine can promote overall improvement in the physical functions of patients. Compared to Western medicine, TCM generally has fewer side effects and is better tolerated by children, making it more suitable for long-term treatment. However, the distinctive smell of Chinese herbal medicine preparations may negatively impact children’s compliance with oral administration [8,159].

### 4.3. Comprehensive Therapy

Related studies indicate that comprehensive therapy may enhance treatment efficacy [97,98,99]. For example, Liu et al. [97] demonstrated that Tuina (Chinese therapeutic massage) combined with acupuncture could improve behavioral scale scores in autistic patients, with its efficacy surpassing that of acupuncture alone. Fan et al. [98] showed that acupuncture combined with Chinese herbal medicine and modern rehabilitation therapy had significantly better outcomes than rehabilitation therapy alone. Li et al. [99] applied a combination approach of Chinese herbal medicine, acupuncture, and Tuina, effectively improving clinical symptoms and enhancing patients’ language and motor abilities.

It should be noted that children whose language systems are not fully developed may not be able to accurately express discomfort experienced during treatment. Furthermore, their delicate skin and lower tolerance may increase the risk of injury [163]. Therefore, during interventions, it is essential to strictly adhere to professional guidelines based on the specific conditions of the patients to ensure both efficacy and safety.

### 4.4. Advantages, Limitations, and Development Directions

TCM integrates holistic treatment principles and syndrome differentiation [154,155]. It requires high-level professional skills from practitioners, and there is currently no international standardized acupuncture protocol [156]. The unique smell of herbal formulations may reduce treatment adherence in children; thus, innovating Chinese medicine to improve medication adherence is necessary. Future cross-cultural studies should develop explanatory frameworks that adapt to different cultural backgrounds, promoting international standardization. This includes aspects such as acupoints, acupuncture techniques, needle retention time, and stimulation frequency.

## 5. Neuromodulation Technique

**Figure 2:**
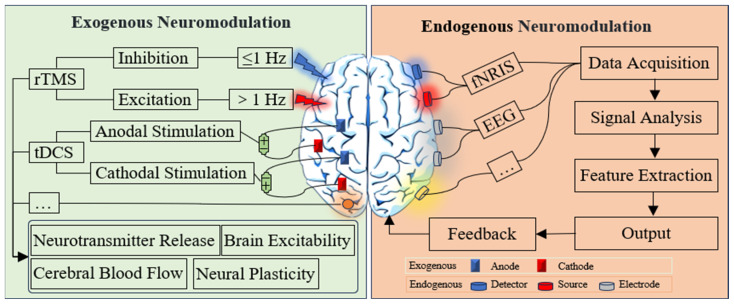
Neuromodulation Technique.

Neuromodulation techniques aim to improve ASD symptoms by modulating neural system activities, and are divided into exogenous neuromodulation technologies (passive approach) and endogenous neuromodulation technologies (active approach), as shown in Figure 2. Exogenous neuromodulation techniques, such as repetitive transcranial magnetic stimulation (rTMS) [100,164,165] and transcranial direct current stimulation (tDCS) [101,166,167,168], regulate cortical excitability by applying magnetic or electric fields to specific brain regions, thereby improving the behavioral symptoms of individuals with ASD. In these techniques, patients passively receive regulation. Endogenous neuromodulation techniques, such as neurofeedback, improve related symptoms through self-regulation, inducing neuroplastic changes [169]. This approach emphasizes the active participation of patients in the regulation process.

### 5.1. Exogenous Neuromodulation Technology

Research has confirmed that targeted stimulation of the unilateral or bilateral dorsolateral prefrontal cortex (DLPFC) can improve executive function and emotional regulation [164]. Stimulating the temporoparietal junction, posterior superior temporal sulcus, and inferior parietal lobule can enhance social cognition and behavior in individuals with autism [165]. Kang et al. [100] evaluated the therapeutic efficacy of 1 Hz rTMS stimulation on children with ASD. In this study, children with ASD accompanied by intellectual disability underwent neuroregulation for approximately 10 min per session, twice a week, for a total of 18 sessions. Electroencephalogram (EEG) features such as recurrence rate, determinism, and mean diagonal length were used as evaluation indicators. The results showed that after the intervention, the recurrence rate and determinism of the children significantly decreased, and there was a significant improvement in the aberrant behavior checklist scores. These findings suggest that rTMS improves the behavior of children with autism by normalizing brain activity.

tDCS may enhance functional brain connectivity [166], improve behavioral and cognitive symptoms [101], enhance social functioning [167,168], and reduce social withdrawal in individuals with autism [170,171]. Stimulation targets include the DLPFC [164,172], the right temporoparietal junction [173,174], and the supplementary motor area. Hadoush et al. studied the efficacy of bilateral anodal tDCS stimulating the DLPFC and motor cortex in improving ASD symptoms [101]. The tDCS group received bilateral anodal stimulation for 20 min per session, five times a week, for two weeks, while the control group received sham stimulation. The results showed that compared to the control group, the tDCS group had significantly lower scores on the autism treatment evaluation checklist in the subscales of sociability and health/physical/behavior. This study suggests that bilateral anodal tDCS has beneficial therapeutic effects in improving symptoms in children with ASD. Additionally, Amatachaya et al. [102] and Costanzo et al. [103] also pointed out that tDCS could be a promising tool for autism treatment.

In summary, rTMS shows significant potential in improving irritability, repetitive, compulsive, and stereotyped behaviors in autism patients, with good safety and tolerability. However, individual variability in efficacy exists [175]. Compared to rTMS, tDCS is more portable and easier to operate, with a lower incidence of side effects [176,177]. The effectiveness of exogenous modulation methods is influenced by factors such as the stimulation target, intensity, and frequency, and there are no universally accepted treatment parameters yet. Additionally, the long-term effects still need further validation [178], and there are contraindications for patients with ASD who have comorbid epilepsy.

### 5.2. Endogenous Neuromodulation Technology

Endogenous neuromodulation is based on the principles of operant conditioning and neuroplasticity [179]. Neurofeedback guides subjects to self-regulate by real-time monitoring, extraction, and feedback of specific neurophysiological signals, inducing functional or structural neural changes [10,107,180,181]. Neurofeedback can effectively improve attention, cognitive flexibility, social behavior, and adaptive functioning in individuals with autism [105,181,182,183].

Kouijzer et al. explored the beneficial effects of EEG-neurofeedback on the executive functions of children with autism [104]. The intervention group received a standardized ADHD neurofeedback protocol twice a week, for 30 min each session, for a total of 40 sessions. The control group did not receive any intervention during the study period. The study found that after treatment, core symptoms of the intervention group, including social interaction, communication, and stereotyped behavior, significantly improved. Improvements were maintained for a year [105]. These results indicate that EEG-neurofeedback can effectively improve the executive functions of children with ASD, with lasting effects. Additionally, the feasibility of improving autism symptoms using fMRI-neurofeedback [106] and fNIRS-neurofeedback [107] has also been confirmed.

In conclusion, endogenous neuromodulation has shown significant effectiveness in improving clinical symptoms in individuals with autism and demonstrates some long-term maintenance effects [184]. Neurofeedback avoids potential side effects and addiction risks associated with drug treatments. However, most studies have small sample sizes and mainly focus on high-functioning individuals with autism (IQ > 70) [185,186], and the efficacy for low-functioning groups requires further validation.

### 5.3. Advantages, Limitations, and Development Directions

Neuroregulation techniques, based on principles of operant conditioning and neuroplasticity, improve the behavior and cognition of individuals with ASD [164,169,179]. However, the high cost and technical demands of neuroregulation technology limit its widespread use in routine clinical practice. Additionally, standardized treatment protocols have not yet been established, including aspects such as stimulation sites, frequency, and intensity [175,178]. In the future, large sample datasets can be used to construct stimulus-response models, optimize or standardize key treatment parameters, develop personalized training paradigm libraries, and integrate intelligent assessment and auxiliary intervention tools to achieve comprehensive monitoring of the rehabilitation process. This will provide more efficient and precise personalized rehabilitation strategies for individuals with ASD. Moreover, it will lay the foundation for elucidating the rehabilitation mechanisms of ASD.

## 6. Complementary and Alternative Medicine

CAM refers to diagnostic and therapeutic approaches and methods that are outside the mainstream medical system, used to supplement or replace conventional treatment methods [187]. Common CAM methods used for ASD treatment include music therapy (MT), animal-assisted intervention (AAI), and exercise intervention, which improve social interaction and communication abilities of autism by promoting emotional expression, social participation, and sensory integration.

### 6.1. Music Therapy

MT includes improvisational music therapy, family-centered music therapy, group music therapy, and mimic music therapy [188,189,190]. Related studies indicate that MT can enhance attention [108], improve emotional recognition and understanding [109,110], promote social behavior [111,112], and strengthen parent-child relationships [113,114]. For example, Thompson et al. [114] demonstrated that family-centered music therapy significantly improved the socio-emotional functioning and parent-child interaction in children with autism. Lagasse et al. [108] confirmed that group music therapy significantly enhanced joint attention and eye contact in children with autism. Forti et al. [115] showed that sound beam mimic intervention significantly improved the mimic accuracy and sustained social attention in children with autism. Existing reviews indicate that most studies based on music therapy primarily assess efficacy using scales [191,192]. Some studies have shown that music therapy also activates emotional and related reward circuits, including the ventral tegmental area, striatum, amygdala, prefrontal cortex, and orbitofrontal cortex [116]. Structural and functional changes have also been observed in brain regions involved in social communication and emotional skills [117,118].

The efficacy of MT is somewhat controversial. Bieleninik et al. conducted a large, multinational, multicenter randomized controlled trial (*n* = 364) to evaluate the effects of improvisational MT on the broad social communication skills of children with autism [119]. The study found no significant improvement in autism diagnostic observation schedule scores before and after treatment or compared to standard care. Currently, most MT studies have small sample sizes, lack rigorous control group designs, and standardization of parameters [193,194,195].

### 6.2. Animal-Assisted Intervention

Dogs, horses, and dolphins are the most commonly used animals in AAI [196,197,198]. Gabriels et al. [120] demonstrated through a large-scale, 10-week randomized controlled trial (*n* = 116) that horse-assisted therapy significantly improved social cognition, social communication, and expressive language abilities in individuals with autism. Hernández-Espeso et al. [121] showed that a 6-week dolphin-assisted therapy program significantly improved the social and communication skills of individuals with autism. Ben-Tzchak et al. [122] found that a four-month dog-assisted intervention significantly enhanced social, communication, and motor skills in individuals with autism. Although most AAI studies reported positive effects on social and communication skills in individuals with autism, the impact on restrictive and repetitive behaviors was generally not significant [123,124].

### 6.3. Exercise Intervention

Exercise interventions have gained widespread attention in autism treatment [199,200,201,202] due to their low cost, ease of implementation, and high acceptance [203], though the underlying neurobiological mechanisms remain unclear [125,200]. Current research indicates that physical activities [125,126] such as swimming, jogging, soccer, and yoga can help improve stereotyped behaviors [127,128], social interaction [129,130,131,132], cognitive flexibility [133,134], and sleep disorders [135,136] in individuals with autism. Sandplay therapy can improve social communication deficits [137] and social interaction [138,139] in children with autism. Moreover, art therapies such as painting, clay modeling, and drama show potential benefits in autism treatment [140,141,142].

### 6.4. Advantages, Limitations, and Development Directions

CAM involves multi-sensory integration and shows considerable potential in alleviating core symptoms and comorbidities of ASD [187]. CAM is characterized by low risk and patient-friendliness, further enriching the intervention techniques for ASD. Future research should consider integrating neuroimaging technologies to explore the potential neural mechanisms and provide empirical support for the design of scientifically effective intervention programs.

## 7. Discussion

Currently, there is no consensus on the etiology and pathogenesis of ASD, and effective biological markers are lacking. Moreover, the heterogeneity of the disorder is significant, with severity ranging from mild to severe, often accompanied by various functional impairments or deficits, which notably affects the formulation of treatment intervention plans and results in unsatisfactory efficacy. This review systematically summarizes and analyzes five types of ASD treatments, each based on different theoretical mechanisms, with varying advantages and disadvantages in addressing the core symptoms and comorbidities of ASD. Similarly, these intervention methods share some common issues in research. For example, they lack standardized treatment protocols, have considerable variability in treatment parameters, involve small sample sizes, and use inconsistent evaluation metrics, limiting comparability and reproducibility. Additionally, efficacy assessments often rely on scales, which are subjective and lack objective, quantitative physiological evaluation indicators. Moreover, most studies focus on short-term efficacy, and the long-term effects remain to be thoroughly investigated. The quantitative relationships between the mechanisms of various intervention methods and the neurobiological basis of ASD (such as abnormal neural connectivity patterns or excitation-inhibition imbalance) have not been fully elucidated.

Due to the lack of specific treatment methods, behavioral interventions and education remain the primary approaches for managing ASD, aiming to maximize the child’s self-care abilities and quality of life while also promoting social skills and communication, leveraging individual strengths, and reducing disability and comorbidity rates. Early intervention is a fundamental principle. Currently, the various popular intervention measures are of varying quality, and there are very few effective interventions proven through evidence-based practice (EBP), leading to ongoing controversies in efficacy evaluation. Treatment for ASD primarily relies on behavioral interventions and education, supplemented by symptomatic medication.

There are numerous behavioral intervention techniques, but they must adhere to individualized, multidimensional, and multidisciplinary approaches, taking into account the reliability of the evidence supporting the interventions. Methods proven effective through EBP should be prioritized, combined with various approaches for comprehensive intervention, and should be flexibly implemented with a focus on specific aspects as necessary. Currently, the interventions with EBP support mainly consist of behavioral therapies. There are no medications available that can improve the core symptoms of ASD; instead, medications are primarily used to address accompanying behavioral and emotional disorders. Pharmacological treatment must weigh the benefits and risks to make optimal clinical decisions. Other popular methods, such as neuroregulation, acupuncture, and CAM, which lack EBP support, have not yet met the standards for clinical promotion and application.

We recommend conducting large-sample, multi-center, and long-term studies in the future to identify critical intervention windows at different developmental stages, systematically evaluate the long-term impacts of various intervention methods on cognitive, behavioral, and other aspects, and provide a basis for precision treatment. Secondly, develop precision intervention pathways and models and construct personalized intervention decision models based on clinical characteristics, neurophenotypes, and environmental factors as well as develop dynamic adjustment mechanisms to optimize intervention plans at different stages of rehabilitation. Thirdly, establish a systematic intervention effect evaluation framework, strengthen the exploration and validation of quantitative biomarkers to improve the objectivity and accuracy of outcome assessments, and facilitate meta-analyses and cross-study comparisons. Additionally, include comorbid symptoms in the evaluation framework to examine their interactions with core ASD symptoms. Finally, establish data-sharing platforms to study the applicability of different intervention methods in various cultural contexts, adjust optimal treatment plans to align with local values and resource availability, and construct a more inclusive and effective global ASD intervention system.

## Figures and Tables

**Table 1:** Summary of intervention methods for ASD.

Treatment Methods	Classification	References	Advantages	Limitations
Pharmacological treatment	Atypical antipsychotic	[6,15,16,17,37,38,39,40,41,42]	Fast-acting, suitable for individuals with severe behavioral issues.	Limited improvement in core symptoms. Potential side effects.
	Stimulant	[6,20,21,48]		
	Antidepressant	[23,24,25,50,51,52,53,54,55]		
	Other medications	[26,27,29,30,31,59,60,61,62,63,64,65,66]		
Behavioral interventions	ABA	[67,68,69,70,71,72,73,74]	Structured training programs that can be adjusted according to individual needs. The cornerstone of early intervention.	Long training periods. Significant individual variability in training outcomes. Effectiveness depends on the professional level of the therapists and the intensity of the intervention.
	NDBIs	[75,76,77,78,79,80,81,82]		
TCM	Acupuncture	[4,83,84,85,86,87,88,89,90,91,92,93]	Low cost. No serious side effects.	Requires practitioners to have a high level of professional skills. No internationally standardized training program. The unique smell of traditional Chinese medicine may reduce patients’ treatment adherence.
	Chinese herbal medicine	[94,95,96]		
	Comprehensive therapy	[97,98,99]		
Neuroregulation	Exogenous neuromodulation	[100,101,102,103]	Passive regulation allows for precise targeting. Active regulation involves high participant engagement and personalization.	High cost. High technical requirements. No standardized treatment protocols.
	Endogenous neuromodulation	[104,105,106,107]		
CAM	Music therapy	[108,109,110,111,112,113,114,115,116,117,118,119]	Low risk and patient-friendly.	Individual differences and personalized needs.
	Animal-assisted intervention	[120,121,122,123,124]		
	Exercise intervention	[125,126,127,128,129,130,131,132,133,134,135,136,137,138,139,140,141,142]		

## Data Availability

No new data were created or analyzed in this study. Data sharing is not applicable to this article.
